# Evaluation of the efficacy of novel topical antifungal agents against dermatophytes in North India: A prospective study

**DOI:** 10.22034/cmm.2024.345268.1562

**Published:** 2024-11-15

**Authors:** Tanya Sachan, Prashant Gupta, Swastika Suvirya, Parul Verma, Raj Kumar Kalyan, Gopa Banerjee

**Affiliations:** 1 Department of Microbiology, King George’s Medical University, Lucknow, Uttar Pradesh, India; 2 Department of Dermatology, Venerology, and Leprosy, King George’s Medical University, Lucknow, Uttar Pradesh, India

**Keywords:** Antifungal Susceptibility, Antifungals, Dermatophytes, ECV, Topical

## Abstract

**Background and Purpose:**

Dermatophytosis, a fungal infection targeting keratinized tissue, is caused by dermatophytes, commonly affecting skin, hair, and nails. Prevalent in tropical regions, such as India, its treatment typically utilizes systemic and topical antifungal medications. Despite ample research on oral antifungals, data on the susceptibility of topical treatments, especially in India, where they are prevalent, remains scarce. This study aimed to investigate the antifungal susceptibility of efinaconazole, tavaborole, luliconazole, and sertaconazole against dermatophytes isolated from cases of dermatophytosis.

**Materials and Methods:**

Samples of all the clinically diagnosed cases of dermatophytosis were subjected to microscopy and culture. All 204 dermatophytes, namely *Trichophyton rubrum* (n=90), *Trichophyton mentagrophytes/interdigitale* (n=69), *Trichophyton tonsurans* (n=44), and *Epidermophyton floccosum* (n=1) were subjected to antifungal susceptibility testing for efinaconazole, tavaborole, sertaconazole, and luliconazole per Clinical Laboratory Standards Institute broth microdilution method (M38-A3).

**Results:**

The minimum inhibitory concentration values for efinaconazole, tavaborole, sertaconazole, and luliconazole were within the ranges of 0.008-0.5, 1-2, 0.128-2, and 0.004-0.008 µg/ml, respectively across all dermatophytes. Epidemiological cutoff values (ECVs) were 0.004 µg/ml for luliconazole and 2 µg/ml for tavaborole for all dermatophytes. Sertaconazole ECVs were 2 µg/ml for *T. rubrum* and *T. mentagrophytes/interdigitale*, 0.5 µg/ml for *T. tonsurans*, and 1 µg/ml for *E. floccosum*. Tavaborole ECVs for *T. mentagrophytes/interdigitale*, *T. tonsurans*, *T. rubrum*, and *E. floccosum* were 0.5, 0.5, 0.25, and 0.016 µg/ml, respectively.

**Conclusion:**

The results from the present study on the *in vitro* performance of newer topical antifungals suggested that they hold significant promise as prospective candidates for advancing the development of new antifungal treatments for dermatophytosis.

## Introduction

Dermatophytes are a group of aerobic fungi known for their ability to produce proteases, enzymes that facilitate the breakdown of keratin. This unique capability enables them to effectively colonize, invade, and cause infections in the outermost layer of the skin (stratum corneum), the hair shaft, and nails [ 1 ]. Mycoses are broadly divided into three types, namely superficial mycoses, subcutaneous mycoses, and systemic mycoses [ 2 ]. Superficial dermatophytosis, impacting 20-25% of the global population, has evolved into a prevalent infectious dermatosis in clinical settings [ 3 ]. It was initially perceived as a mild and easily treatable infection prevalent in tropical and subtropical regions during summer and rainy seasons. However, it has now transformed into a persistent and challenging entity in India. Recent studies have highlighted a notable rise in dermatophytosis incidence nationwide, particularly over the past decade and markedly so in the last 5-6 years. Studies from South India have reported a prevalence rate of 6.09-27.6%. In contrast, North India has witnessed a notably higher prevalence rate, reaching as high as 61.5%, emphasizing regional disparities in the prevalence of the condition [ 3 ].

In recent years, there has been a notable neglect in researching dermatophytosis treatment, despite the escalating global prevalence, especially in tropical regions, which is a cause for concern. Various treatment modalities are available for the management of dermatophytosis, with oral antifungals being the mainstay of treatment. While these medications show promising clinical cure rates, they come with significant limitations, including the emergence of antifungal drug resistance and the potential for adverse reactions. Hence, there is a need for potent topical antifungals to bypass these concerns. ECTODERM India has also recommended that topical azoles should be the empiric agent of choice in the management of naive and recalcitrant cases [ 4 ].

The treatment landscape for dermatophytosis has seen numerous recent advancements ranging from updated dosing protocols to the introduction of novel drugs. In 2013, the U.S. Food and Drug Administration (USFDA) granted approval to luliconazole cream, 1%, marking a significant milestone in the topical treatment of interdigital tinea pedis, tinea cruris, and tinea corporis caused by *Trichophyton rubrum* and *Epidermophyton floccosum*, specifically in patients aged 18 years and older [ 5 ].

Sertaconazole belongs to the class of imidazole antifungal agents. This medication acts by inhibiting the synthesis of ergosterol, a crucial component in the cell wall of fungi. It has broad-spectrum antifungal activity against dermatophytes of the *Trichophyton*, *Epidermophyton*, and *Microsporum* genera [ 6 ].

Efinaconazole is the first FDA-approved azole in the USA to be used topically in the treatment of dermatophytic onychomycosis. It acts by inhibiting fungal lanosterol 14α-demethylase within the ergosterol biosynthesis pathway, demonstrating potent antifungal activity against dermatophytes [ 7 ].

In addition to the use of azoles, a more recent class of antifungal drugs has gained popularity, known as oxaboroles. Tavaborole stands out as the pioneer in this class. Tavaborole, a boron-containing topical antifungal, received USFDA approval for dermatophytic onychomycosis in 2014. It works by inhibiting leucyl-transfer RNA synthetase, a key enzyme in fungal protein synthesis. Notably, tavaborole eliminates the need for nail debridement and has minimal impact on cytochrome P450 enzymes, reducing potential drug interactions [ 6 ].

Additionally, the Nail Society of India (2023) has recently published its recommendations for the treatment of dermatophytic onychomycosis, which has opened the doors for clinicians to use these newer drugs in the treatment regime [ 8 ].

Given the evolving nature of dermatophyte infections, which now manifest as chronic, unresponsive to treatment, and recurrent cases, the present study aimed to isolate and identify dermatophytes and perform antifungal susceptibility testing for these newer topical antifungal medications.

## Materials and Methods

The present study was conducted in the Department of Microbiology over a period of 12 months. The Institutional Ethics Committee approved the study (Ref. Code: XIV-PGTSC-IIA/P72). In this prospective observational study, clinical specimens were collected from patients with a history of hair, skin, or nail lesions and those recently diagnosed with dermatophytosis. After a thorough study of case history and examination conducted in good lighting, skin scraping, nail clipping, and epilated hair were obtained for analysis. All the samples were subjected to direct microscopic examination with 20-40% potassium hydroxide and fungal culture on Sabouraud Dextrose Agar (SDA) and dermatophyte test media (DTM). Pure isolates were generated by subculturing on SDA and potato dextrose agar (PDA) media for microscopic examination of culture and morphological characteristics for further differentiation, respectively. Sub-cultures on PDA were used for identification by matrix-assisted laser desorption ionization-time of flight mass spectrometry (MALDI-TOF MS [Vitek MS]) and antifungal susceptibility testing. All the isolates were identified by MALDI-TOF MS and conventional methods collectively. Both methods were compared, and agreement was calculated. Antifungal susceptibility was performed for luliconazole, sertaconazole, efinaconazole, and tavaborole (Cayman Chemical-USA) using the broth microdilution method as per Clinical Laboratory Standards Institute (CLSI) M-38 A3 guidelines for moulds to determine the minimum inhibitory concentration (MIC) values [ 9 ]. An initial concentration of 200 µg/ml (stock) was prepared, which was further diluted to get the final concentration of medications. Epidemiological cutoff values (ECVs) were calculated; for this purpose, the MIC that encompassed ≥ 97.5% of all MIC values in the distribution was designated the ECV [ 10 ]. The MIC_50_ and MIC_90_ values of isolates were also recorded.

### Statistical analysis

For the prevalence rate of dermatophyte fungal infection, which was 53.4%, a sample size of 383 was selected within a 5% error margin and with a 95% confidence interval [ 11 ]. The collected data were entered into the Excel software, and analysis was conducted in SPSS software (version 26). Continuous variables are summarised descriptively in the tables.

## Results

**Figure 1:**
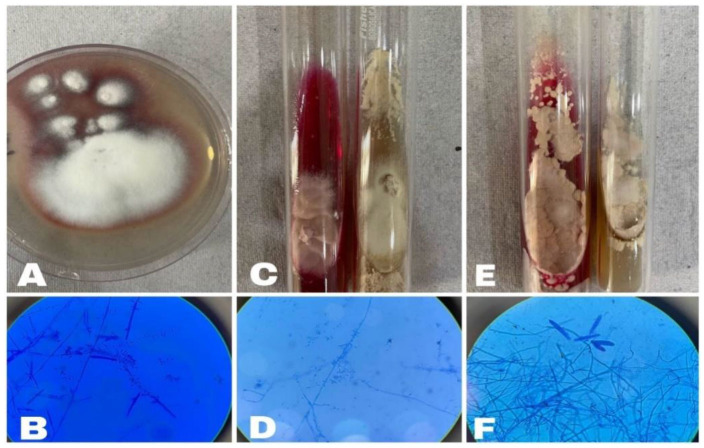
A: *Trichophyton rubrum* on potato dextrose agar (PDA): fluffy, white buff colony on the obverse, B: Lactophenol cotton blue (LPCB) stain showing *Trichophyton rubrum*: MICROCONIDIA: tear-shaped found singly all along hyphae. MACROCONIDIA: Long narrow, pencil-like, C: *Trichophyton tonsurans* on dermatophyte test media (DTM) and Sabouraud’s Dextrose Agar: Powdery to cream/yellow with central furrows, D: LPCB stain showing tear drop-shaped microconidia of *Trichophyton tonsurans*, E: *Trichophyton mentagrophytes* on DTM and PDA: surface buff and powdery, F: LPCB stain showing cigar-shaped, thin-walled microconidia of *Trichophyton mentagrophytes*

In total, 445 clinically suspected cases of dermatophytosis who attended the Dermatology Outpatient Department (OPD) were included in this study, and 204 of them were diagnosed with dermatophytes. The majority (45.09%) of the total enrolled patients had dermatophytic onychomycosis, followed by tinea corporis (32.3%), tinea cruris (7.84%), tinea manuum (7.84%), tinea capitis (2.4%), tinea pedis (1.96%), tinea faciei (1.47%), and tinea barbae (0.98%). Moreover, 93.6%, 90.2%, and 98.03% of the isolates were positive on KOH microscopy, SDA culture, and DTM culture, respectively. Considering DTM culture as the gold standard, diagnostic parameters were calculated of KOH microscopy. Overall sensitivity and specificity values of KOH microscopy based on DTM culture were 94% and 75%, respectively (Table 1). Based on the results, 196 out of 204 dermatophytes could be identified by MALDI-TOF MS, and the remaining 8 were processed based on identification by conventional methods. The identification rates by MALDI-TOF MS for *Trichophyton mentagrophytes/interdigitale*, *Trichophyton rubrum*, and *Trichophyton tonsurans* were 98.5%, 97.8%, and 90.7%, respectively. The isolates that could not be identified by MALDI-TOF MS included 1 *Epidermophyton floccosum*, 1 *T. mentagrophytes /interdigitale*, 2 *T. rubrum*, and 4 *T. tonsurans*. The agreement between conventional and MALDI-TOF MS was seen in 95.1% of isolates, which was statistically significant (*P*<0.001). The most frequently isolated dermatophyte was caused by *T. rubrum* (44.1%; 90/204), followed by *T. mentagrophytes/interdigitale* (33.8%; 69/204), *T. tonsurans* (21.6%; 44/204), and *E. floccosum* (0.5%; 1/204) (Figure 1). The MIC range for all dermatophytes was the lowest for luliconazole (0.004-0.008 µg/ml). The majority of isolates of *T. rubrum*, *T. mentagrophytes/interdigitale*, *T. tonsurans*, and *E. floccosum* showed the lowest MIC tested for luliconazole, which was 0.004 µg/ml (Table 2,3). For sertaconazole, the MIC ranged from 0.128 to 2 µg/ml for all isolates. Maximum isolates of *T. rubrum* (68) and *T. mentagrophytes/interdigitale* (43) exhibited an MIC of 0.5 µg/ml. In contrast, for *T. tonsurans* (36), the maximum isolates showed a MIC of 0.250 µg/ml (Table 3, 4). The MIC values for all dermatophytes for efinaconazole ranged from 0.008 to 0.5 µg/ml, with the maximum isolates of *T. rubrum* (68), *T. mentagrophytes /interdigitale* (54), *T. tonsurans* (30), and *E. floccosum* (1) having a MIC of 0.016 µg/ml (Tables 5, 3). Tavaborole demonstrated MIC values ranging from 1 to 2 µg/ml across all isolates. The majority of *T. rubrum isolates* (63), *T. mentagrophytes/interdigitale* (58), *T. tonsurans* (28), and *E. floccosum* (1) exhibited an MIC of 2 µg/ml (Tables 6, 3). The ECV, MIC_50_, and MIC_90_ values for luliconazole, sertaconazole, efinaconazole, and tavaborole against various species of dermatophytes are enumerated in Table 7.

**Table 1:** Diagnostic parameters of KOH microscopy considering Dermatophyte Test Media culture as the gold standard

	DTM Positive(n=200)	DTM Negative(n=4)	
KOH positive	188 (TP)	3 (FP)	Positive predictive value (98.4%)
KOH negative	12 (FN)	1 (TN)	Negative predictive value (92.3%)
	Sensitivity (94%)	Specificity(75%)	

DTM: dermatophyte test media, TP: True Positive, FP: False Positive, FN: False Negative, TN: True Negative

**Table 2:** Minimum inhibitory concentration distribution of luliconazole for dermatophytes

Species	Total isolates (n=204)	MIC (μg/ml) values of the isolates (n)									
		0.004	0.008	0.016	0.032	0.064	0.128	0.25	0.5	1	2
*Trichophyton rubrum*	90	88	2	-	-	-	-	-	-	-	-
*Trichophyton mentagrophytes /interdigitale*	69	67	2	-	-	-	-	-	-	-	-
*Trichophyton tonsurans*	44	44	-	-	-	-	-	-	-	-	-
*Epidermophyton flocossum*	1	1	-	-	-	-	-	-	-	-	-

MIC: minimum inhibitory concentration

**Table 3:** Minimum inhibitory concentration distribution of Sertaconazole for dermatophytes

Species	Total isolates (n=204)	MIC (µg/ml) values of the isolates (n)									
		0.004	0.008	0.016	0.032	0.064	0.128	0.25	0.5	1	2
*Trichophyton rubrum*	90	-	-	-	-	-	9	3	68	-	10
*Trichophyton mentagrophytes /interdigitale*	69	-	-	-	-	-	5	-	43	15	6
*Trichophyton tonsurans*	44	-	-	-	-	-	-	36	8	-	-
*Epidermophyton flocossum*	1	-	-	-	-	-	-	-	-	1	-

MIC: minimum inhibitory concentration

**Table 4:** Minimum inhibitory concentration distribution of efinaconazole for dermatophytes

Species	Total isolates (n=204)	MIC (µg/ml) values of the isolates (n)									
		0.004	0.008	0.016	0.032	0.064	0.128	0.25	0.5	1	2
*Trichophyton rubrum*	90	-	6	68	3	3	2	6	2	-	-
*Trichophyton mentagrophytes /interdigitale*	69	-	-	54	4	3	2	1	5	-	-
*Trichophyton tonsurans*	44	-	-	30	4	-	-	-	10	-	-
*Epidermophyton flocossum*	1	-	-	1	-	-	-	-	-	-	-

MIC: minimum inhibitory concentration

**Table 5:** Minimum inhibitory concentration distribution of Tavaborole for dermatophytes

Species	Total isolates (n=204)	MIC (µg/ml) values of the isolates (n)									
		0.004	0.008	0.016	0.032	0.064	0.128	0.25	0.5	1	2
*Trichophyton rubrum*	90	-	-	-	-	-	-	-	-	27	63
*Trichophyton mentagrophytes /interdigitale*	69	-	-	-	-	-	-	-	-	11	58
*Trichophyton tonsurans*	44	-	-	-	-	-	-	-	-	16	28
*Epidermophyton flocossum*	1	-	-	-	-	-	-	-	-	-	1

MIC: minimum inhibitory concentration

**Table 6:** Epidemiological Cutoff Values (ECVs) and percentage of isolates above ECV for luliconazole, sertaconazole, efinaconazole and tavaborole for dermatophyte species

Species	Antifungal agent	ECV (µg/ml)	Percentage of isolates above ECV
Luliconazole	*Trichophyton rubrum*	0.004	2.22
	*Trichophyton mentagrophytes/interdigitale*	0.004	2.89
	*Trichophyton tonsurans*	0.004	0
	*Epidermophyton flocossum*	0.004	0
Sertaconazole	*Trichophyton rubrum*	2	0
	*Trichophyton mentagrophytes/interdigitale*	2	0
	*Trichophyton tonsuran*	0.5	0
	*Epidermophyton flocossum*	1	0
*Efinaconazole*	*Trichophyton rubru*	0.25	2.22
	*Trichophyton mentagrophytes/interdigital*	0.5	0
	*Trichophyton tonsurans*	0.5	0
	*Epidermophyton flocossum*	0.016	0
*Tavaborole*	*Trichophyton rubrum*	2	0
	*Trichophyton mentagrophytes/interdigitale*	2	0
	*Trichophyton tonsurans*	2	0
	*Epidermophyton flocossum*	2	0

**Table 7:** Range, geometric mean, mode, MIC_50_, and MIC_90_ for tavaborole, efinaconazole, sertaconazole and luliconazole for dermatophyte species by the CLSI M38-A3 broth microdilution method

Species	Antifungal agent	Range (µg/ml)	Geometric mean	MIC Mode (µg/ml)	MIC_50_ (µg/ml)	MIC_90_ (µg/ml)
*Epidermophyton floccosum* (n=1)	Tavaborole2-2	2	2	2	2
	Efinaconazole	0.016-0.016	0.016	0.016	0.016	0.016
	Sertaconazole	1.0-1.0	1.0	1	1	1
	Luliconazole	0.004-0.004	0.004	0.004	0.004	0.004
*Trichophyton mentagrophytes/interdigitale* (n=69)	Tavaborole	1-2	1.79	2	2	2
	Efinaconazole	0.016-0.50	0.025	0.016	0.016	0.128
	Sertaconazole	0.128-2.00	0.594	0.5	0.5	1
	Luliconazole	0.004-0.008	0.004	0.004	0.004	0.004
*Trichophyton rubrum* (n=90)	Tavaborole	1-2	1.62	2	2	2
	Efinaconazole	0.008-0.50	0.022	0.016	0.016	0.128
	Sertaconazole	0.128-2.00	0.497	0.5	0.5	2
	Luliconazole	0.004-0.008	0.004	0.004	0.004	0.004
*Trichophyton tonsurans* (n=44)	Tavaborole	1-2	1.55	2	2	2
	Efinaconazole	0.016-0.50	0.037	0.016	0.016	0.5
	Sertaconazole	0.250-0.50	0.284	0.25	0.25	0.5
	Luliconazole	0.004-0.004	0.004	0.004	0.004	0.004

MIC: minimum inhibitory concentration, ECV: epidemiological cutoff value

## Discussion

Dermatophytosis poses a significant public health challenge in tropical and subtropical regions, such as India, persisting as an ongoing issue. Given the escalating incidence rate of recalcitrant and resistant cases of dermatophytosis, there is an urgent need for swift and accurate identification of the causative fungi and the conduction of antifungal susceptibility testing.

In this study, the most commonly encountered dermatophytic infection was dermatophytic onychomycosis (45.09%). This is in contrast with most of the literature to date where the prevalence of tinea corporis was found to be maximum. Studies performed by Hosthota et al., Verma et al., Singh et al., and Karmakar et al. have shown relatively lower incidence rates of 4.2%, 4%, 1.9%, and 2.8% of dermatophytic onychomycosis, respectively [ 12 - 15 ]. This variation could be attributed to the predominant age group and other associated systemic co-morbidities, like diabetes [ 16 ]. 

In the present study, five patients were noted to have diabetes mellitus as a risk factor and three of them suffered from dermatophytic onychomycosis. Diabetes is an established risk factor for onychomycosis. In a study conducted by Agarwal in north India, the prevalence rate of tinea unguium among diabetic patients was found to be 34% [ 17 ]. Moreover, most of the patients presenting with onychomycosis were farmers and laborers; continuous contact of toenails with soil and repeated trauma could also be among the risk factors. Tinea corporis was seen in 32.3% of total cases in the present study which mirrors the studies carried out by Singh et al., Bindu et al., Noronha et al., and Vinitha et al. They reported the prevalence rates of tinea corporis between 31.2% and 48.7% [ 18 - 21 ].

In this study, 96.07% of the total isolates could be identified by MALDI-TOF-MS which matches with the results published by Azrad et al. In their study, they obtained correct identification for 87% of the isolates [ 22 ].

In the present research, *T. rubrum* (44.1%) emerged as the most commonly isolated species, followed by *T. mentagrophytes/interdigitale* (33.8%), *T. tonsurans* (21.6%), and *E. floccosum* (0.5%). Similar results were demonstrated in a study conducted by Patel et al., wherein *T. rubrum* comprised 57.4% of the total isolates [ 23 ]. Predominance of *T. rubrum* has also been reported by several other studies across India [ 24 ].

Notably, the MIC range for all dermatophytes in this study was lowest for luliconazole (0.004-0.008 µg/ml), which is consistent with the findings of a study performed by Matehkolaei et al. [ 25 ]. The majority of isolates of *T. rubrum*, *T. mentagrophytes/interdigitale*, *T. tonsurans*, and *E. floccosum* exhibited the lowest MIC tested, at 0.004 µg/ml, stating the broad-spectrum activity of luliconazole against all the dermatophyte species. Similarly, Tahiliani et al. have also reported the lowest mean MIC values for luliconazole among all the topical antifungals tested [ 26 ].

Furthermore, epidemiological cutoff values for luliconazole indicated that the majority of isolates were equal to or lower than 0.004 µg/ml, indicating that relatively lower concentrations of the drug are required for its activity against dermatophyte isolates.

Moreover, Rana et al. have demonstrated the superiority of topical luliconazole over bland emollients (a mixture of mineral oils and humectants, like glycerin and hyaluronic acid) when given as an adjuvant with oral itraconazole. Cure rates of 82.35% were found with this combination, which further adds to the utility of topical luliconazole in the management of dermatophytosis [ 27 ].

Sertaconazole is a broad-spectrum imidazole antifungal agent that has been utilized for the treatment of various skin infections caused by dermatophytes. 

Several studies have demonstrated the potent activity of sertaconazole against the most common dermatophytes, including *T. rubrum*, *T. mentagrophytes*, and *E. floccosum*. The MICs for sertaconazole were within the range of 0.128-2 µg/ml, which was similar to the findings of Rudramurthy et al. [ 28 ]. Carrillo-Muñoz et al. conducted a study using a broth microdilution method to test 53 strains of dermatophytes. They reported the following rank order of potency: terbinafine, followed by sertaconazole and bifonazole [ 29 ]. Although terbinafine demonstrates excellent activity *in vitro*, the emergence of resistance is becoming a significant clinical issue. Numerous cases of terbinafine-resistant dermatophytosis caused by a newly identified species within the *T. mentagrophytes/interdigitale* complex have been reported in India. Initially classified as either *T. interdigitale* or *T. mentagrophytes* type VIII, this taxon is now recognized as a distinct species called *Trichophyton indotineae*. Currently, over 70% of all dermatophyte strains of *T. rubrum* and *T. indotineae* isolated in India exhibit resistance to terbinafine [ 30 ]. Additionally, in another study conducted by Carrillo-Muñoz et al., the efficacy of sertaconazole was shown to be higher in resistant strains, thereby demonstrating its utility in addressing recent infection trends [ 31 ].

Among the two recently USFDA-approved antifungals, efinaconazole exhibited a lower mean MIC value, compared to Tavaborole. In their study, Siu et al. have demonstrated the MIC value of efinaconazole to be 0.001-0.015 µg/ml for *T. rubrum*, 0.001-0.03 µg/ml for *T. mentagrophytes*, 0.016 µg/ml for *T. tonsurans*, and 0.002-0.0078 µg/ml for *E. flocossum*, which is congruent with the findings of the present study [ 32 ]. The MICs for tavaborole were significantly high in the present study, as well as a study performed by Markham et al., ranging between 1-8 µg/ml [ 33 ]. This suggests that efinaconazole tends to be more effective *in vitro* than tavaborole at lower MICs. This finding is consistent with the results reported by Tachibana et al. [ 34 ].

*In vitro* studies have revealed that efinaconazole exhibits potent activity against various dermatophyte species, *T. rubrum*, *T. mentagrophytes*, and *E. flocossum*, akin to other synthesized azoleamine agents. Given that many antifungal medications are hindered in their effectiveness due to keratin binding, the efficacy of efinaconazole against *T. mentagrophytes* was assessed in the presence of keratin. Notably, efinaconazole exhibited less deactivation, compared to its counterparts in the presence of keratin, attributed to its 4-methylenepiperidino group. Furthermore, in a guinea pig model of tinea corporis, efinaconazole demonstrated the highest efficacy among the tested medications against *T. mentagrophytes* and exhibited superior penetration via both trans follicular and trans epidermal routes [ 35 ].

Moreover, tavaborole, despite having higher MICs, is a potential medication in the treatment of dermatophytic onychomycosis as it has greater nail penetration activity and obtains concentrations greater than the MIC of other topical antifungal agents currently in use, which could be attributed to its low molecular weight. In addition, Hui et al. conducted a study to determine the *in vitro* penetration of tavaborole, compared to commercial ciclopirox, and their findings demonstrated the superior activity and pharmacokinetics of tavaborole, compared to ciclopirox [ 36 ].

Another important concept related to antifungal agents is the ECV, which signifies the minimum inhibitory concentration (MIC) value distinguishing microbial populations with and without acquired or mutational resistance based on their phenotypes [ 37 ]. In the present study, only 2.22% of the isolates of *T. rubrum* and 2.89% of the isolates of *T. mentagrophytes/interdigitale* showed a MIC above the ECV for luliconaozle, while none of the isolates showed a greater MIC than ECV for sertaconazole and tavaborole. In their study, Shaw et al. reported that 13.9% of the isolates of *T. mentagrophytes/interdigitale* showed MIC values above the upper limit of wild type, while for sertaconazole this fraction was considerably low (0.2%) [ 38 ]. Although ECV only defines the upper limit of susceptibility for the wild-type population of the microbe and is solely based on *in vitro* laboratory data and also cannot be used by itself to predict the clinical outcome of therapy, it can still be a useful tool in clinical decision-making, especially in cases where the antifungal medication is not responding.

Studies have also shown the mycological cure rates of these newer medications, namely efinaconazole, tavaborole, sertaconazole, and luliconazole, to be at par with the currently available topical treatments [ 34 ]. Several clinical trials have also been conducted to determine the safety of these medications and only a few localized adverse effects have been reported to date. Efinaconazole, tavaborole, sertaconazole, and luliconazole have an additional advantage in that only a negligible amount of the drug is absorbed into the bloodstream; therefore, posing minimum systemic side effects. As a result, the reduced need for monitoring and lower apprehension when utilizing topical antifungal agents suggest that efinaconazole and tavaborole may serve as viable alternatives to oral antifungals for treatment of patients with dermatophytic onychomycosis [ 35 ].

Findings of this study have to be seen in light of some limitations. Since this was an institutional study, the study population might not reflect the true external validity of the results. Therefore, further multicentric research is required to corroborate the findings.

## Conclusion

The findings from the current study regarding the *in vitro* performance of efinaconazole, tavaborole, sertaconazole, and luliconazole indicate that these medications show great potential as a prospective candidate for the advancement of the development of a new antifungal treatment of dermatophytic onychomycosis. Further *in vivo* studies are required to confirm these findings. Moreover, additional research in pharmacokinetics and pharmacodynamics is necessary to establish the MIC breakpoints for these drugs.
